# Differential effects of lesion mimic mutants in barley on disease development by facultative pathogens

**DOI:** 10.1093/jxb/erv154

**Published:** 2015-04-08

**Authors:** Graham R. D. McGrann, , Andrew Steed, Christopher Burt, Paul Nicholson, James K. M. Brown

**Affiliations:** Department of Crop Genetics, John Innes Centre, Norwich Research Park, Norwich, NR4 7UH, UK; ^1^ Present address: Crop Protection Team, Crop and Soil Systems Group, SRUC, West Mains Road, Edinburgh, EH9 3JG, UK; ^2^ Present address: RAGT Seeds Ltd., Grange Road, Ickleton, Essex, CB10 1TA, UK

**Keywords:** Cell death, disease resistance, endophyte, hemibiotroph, hypersensitive response, *mlo*, necrotroph, plant-microbe interactions.

## Abstract

Lesion mimic mutants display spontaneous necrotic spots and chlorotic leaves as a result of mis-regulated cell death programmes. Typically these mutants have increased resistance to biotrophic pathogens but their response to facultative fungi that cause necrotrophic diseases is less well studied. The effect of altered cell death regulation on the development of disease caused by *Ramularia collo*-*cygni*, *Fusarium culmorum* and *Oculimacula yallundae* was explored using a collection of barley necrotic (*nec*) lesion mimic mutants. *nec8* mutants displayed lower levels of all three diseases compared to *nec9* mutants, which had increased *R*. *collo*-*cygni* but decreased *F*. *culmorum* disease symptoms. *nec1* mutants reduced disease development caused by both *R*. *collo*-*cygni* and *F*. *culmorum*. The severity of the *nec1*-induced lesion mimic phenotype and *F. culmorum* symptom development was reduced by mutation of the negative cell death regulator *MLO*. The significant reduction in *R*. *collo*-*cygni* symptoms caused by *nec1* was completely abolished in the presence of the *mlo-5* allele and both symptoms and fungal biomass were greater than in the wild-type. These results indicate that physiological pathways involved in regulation of cell death interact with one another in their effects on different fungal pathogens.

## Highlight

Different pathways that regulate cell death have distinct effects on diverse pathogens. *MLO* and *NEC1*, which both suppress cell death, interact with one another in their effects on pathogenic fungi.

## Introduction

Programmed cell death is essential for many plant developmental processes such as leaf senescence and plays a critical role in defence against pathogens (Jones, 2001). Localized cell death at the sites of pathogen infection is termed the hypersensitive response (HR). HR forms part of the defence response referred to as effector triggered immunity (ETI), which is associated with the production of antimicrobial compounds, cell wall cross-linking, deposition of callose, and a prolonged reactive oxygen species (ROS) burst (Nurnberger *et al.*, 2004; Jones and Dangl, 2006). ETI is particularly effective against pathogens that have a biotrophic lifestyle, requiring living host tissue on which to feed (Glazebrook, 2005; Jones and Dangl, 2006). However, the role of cell death in defence against facultative pathogens that may benefit from, or actively induce host cell death is not as clear. Cell death can operate against some hemibiotrophic pathogens that require a period of biotrophic development before becoming necrotrophic but is not effective against pathogens during the necrotrophic phase (Glazebrook, 2005; Mengiste, 2012).

Mutagenesis of plants resulting in altered disease resistance has proved valuable in dissecting the defence response to different pathogens (Hammond-Kosack and Parker 2003). Lesion mimic mutants develop spontaneous necrotic lesions in the absence of pathogen infection. This phenotype is caused by altered regulation of cell death processes such as HR and senescence or by perturbation of metabolic pathways resulting in cell death (Dangl *et al.*, 1996). Mutations in genes involved in processes such as cellular signalling, chlorophyll biosynthesis, redox homeostasis, and disease resistance can result in lesion mimic phenotypes and these have advanced our understanding of the programmed cell death and HR pathways (Dangl *et al.*, 1996; Lorrain *et al.*, 2003; Moeder & Yoshioka, 2008). As a consequence of the association with cell death, lesion mimic mutants often exhibit accelerated leaf senescence and altered ROS homeostasis (Lorrain *et al.*, 2003). Lesion mimic mutants have been extensively studied in relation to plant defence responses and typically show enhanced resistance against biotrophic pathogens such as rusts and mildews (Kamlofski *et al.*, 2007; Zhang *et al.*, 2009). More variable responses have been reported between lesion mimics and facultative fungi, ranging from enhanced resistance (Persson *et al.*, 2008; 2009) to super-susceptibility (Wright *et al.*, 2013).

One gene of agronomic significance which when mutated in barley causes necrotic lesions is *MLO*. Recessive *mlo* mutations confer broad-spectrum durable resistance to the obligate biotrophic powdery mildew fungus *Blumeria graminis* f. sp. *hordei* and cause developmentally controlled lesion mimic phenotypes in the absence of disease (Wolter *et al.*, 1993). Mutant *MLO* alleles have associated deleterious agronomic effects including reduced yield (Kjaer *et al.*, 1990) and increased susceptibility to some facultative pathogens such as *Fusarium graminearum* (Jansen *et al.*, 2005), *Magnaporthe oryzae* (Jarrosch *et al.*, 1999), *Bipolaris sorokiniana* (Kumar *et al.*, 2001) and *Ramularia collo*-*cygni* (McGrann *et al.*, 2014). MLO encodes a seven-transmembrane domain protein that has been proposed to act as a negative regulator of cell death and disease resistance (Peterhänsel *et al.*, 1997; Piffanelli *et al.*, 2002), but the exact biochemical function of this protein remains undetermined (Buschges *et al.*, 1997).

Necrotic (*nec*) mutants from a fast-neutron exposed barley collection show varying degrees of leaf spotting, chlorosis and in most cases increased expression of HR-induced genes (Rostoks *et al.*, 2003). Genetic analyses of some of these mutants have identified the genes responsible for the lesion mimic phenotype. *nec1* mutants which show reduced basal resistance against powdery mildew fungi and enhanced nonhost resistance against *Pseudomonas syringae* pv. *tomato* (Keisa *et al.*, 2011) have mutations in a cyclic nucleotide-gated ion channel 4 protein (CNGC4; Rostoks *et al.*, 2006). CNGCs are cation channel proteins involved in regulating intracellular fluxes of ions such as Ca^2+^ (Ma and Berkowitz, 2011). These non-selective cation channels have been well studied in the model plant *Arabidopsis thaliana* and function in biological processes including ion homeostasis, development, plant defence and programmed cell death (Ma and Berkowitz, 2011; Moeder *et al.*, 2011). Barley *nec8* mutants show elevated resistance against stem rust (*Puccinia graminis*) but not stripe rust (*P*. *striiformis* f. sp. *hordei*; Zhang *et al.*, 2009). Transcript-based cloning identified a cation/proton exchanging protein as a strong candidate for *nec8* (Zhang *et al.*, 2009) further highlighting the role of mis-regulation cellular cation concentrations in the development of the lesion mimic phenotype, the cell death programme and plant defence responses (Ma and Berkowitz, 2011).

This study examined the response of a collection of barley lesion mimic mutants to facultative fungal pathogens that exhibit different life habits. *Ramularia collo*-*cygni* is an endophytic fungus that under certain environmental conditions becomes a necrotrophic pathogen causing the disease Ramularia leaf spot (RLS; Walters *et al.*, 2008; Havis *et al.*, 2015). *R*. *collo*-*cygni* develops asymptomatically from infected seed (Havis *et al.*, 2014) and from air-borne spore infection (Stabentheiner *et al.*, 2009) with disease symptoms typically occurring at the end of the growing season coincident with a decline in the host antioxidant system as the crop senesces, suggesting that RLS development may be linked to host stress (Schützendübel *et al.*, 2008; McGrann *et al.*, 2015). *Oculimacula yallundae* is a hemibiotrophic pathogen and one of the fungal species responsible for the stem base eyespot disease of cereals. Similar to *R*. *collo*-*cygni*, *O*. *yallundae* has a long period of asymptomatic colonization before the fungus enters the necrotrophic disease-causing phase (Blein *et al.*, 2009). *Fusarium culmorum* is also a hemibiotrophic fungus that causes disease in the ears, stems, leaves and roots of cereal plants (Scherm *et al.*, 2013). Post inoculation disease symptoms form rapidly following a short period of biotrophic development, with disease lesions visible within a few days (Chen *et al.*, 2009). The data reported here shows that symptom development of these three diseases is differentially affected in barley *nec* mutants. Furthermore, this data highlights a previously unreported functional relationship between the *NEC1* and *MLO* genes in the regulation of plant-pathogen interactions and programmed cell death pathways.

## Material and methods

### Plant material

Details of the barley lesion mimic mutants used in this study are shown in Table 1. Seeds were sown in 8×8×10cm pots containing Levington F2 compost media (Scotts Professional, Ipswich, UK). Plants were grown in a controlled environment room (Sanyo) with a day/night photoperiod of 16h/8h at temperatures of 18/12°C supplemented with 220 μmol m^-2^ s^-1^ fluorescent lighting before and after inoculation.

**Table 1.** Barley lesion mimic mutants used in this study

Line	Mutation	Mutagen	Background	Seed source	Reference
Steptoe	None	n/a	Wild-type	n/a	
Morex	None	n/a	Wild-type	n/a	
FN044	*nec8*	Fast neutron	Steptoe	M2-selection	Zhang *et al.* (2009)
FN085	*nec1*	Fast neutron	Steptoe	M2-selection	Rostoks *et al*. (2003, 2006)
FN093	Unknown	Fast neutron	Steptoe	M2-selection	Rostoks *et al.* (2003)
FN211	*nec8*	Fast neutron	Steptoe	M2-selection	Zhang *et al.* (2009)
FN227	*nec9*	Fast neutron	Steptoe	M2-selection	Rostoks *et al.* (2003)
FN303	*nec8*	Fast neutron	Steptoe	M2-selection	Zhang *et al.* (2009)
FN338	*nec1*	Fast neutron	Morex	M2-selection	Rostoks *et al*. (2003, 2006)
FN364	*nec9*	Fast neutron	Steptoe	M2-selection	Rostoks *et al.* (2003)
FN366	Unknown	Fast neutron	Steptoe	M2-selection	Rostoks *et al.* (2003)
FN367	Unknown	Fast neutron	Steptoe	M2-selection	Rostoks *et al.* (2003)
FN370	*nec1*	Fast neutron	Steptoe	M2-selection	Rostoks *et al*. (2003, 2006)
FN450	*nec9*	Fast neutron	Steptoe	M2-selection	Rostoks *et al.* (2003)
FN451	Unknown	Fast neutron	Steptoe	M2-selection	Rostoks *et al.* (2003)
Parkland (G10-30)	None	n/a	Wild-type	n/a	Keisa *et al.* (2011)
GSH01284 (G10-29)	*nec1*	Spontaneous	Parkland	n/a	Keisa *et al.* (2011)
G10-31	*nec1*+*mlo5*	Spontaneous + ethyl methanesulfonate	GSH01284 × Carlsberg II	F4 family	Keisa *et al.* (2011)
G10-32	*nec1*	Spontaneous	GSH01284 × Carlsberg II	F4 family	Keisa *et al.* (2011)
G10-34	None	n/a	GSH01284 × Carlsberg II	F4 family	Keisa *et al.* (2011)
G10-36	*mlo5*	Ethyl methanesulfonate	GSH01284 × Carlsberg II	F4 family	Keisa *et al.* (2011)

### *Ramularia collo-cygni* inoculation

Fourteen-day-old prophyll leaves were inoculated with a liquid inoculum containing macerated hyphal fragments of *R*. *collo*-*cygni* isolate Rcc09B4 based on the method of Makepeace *et al.* (2008) with the modifications outlined in Peraldi *et al.* (2014). Disease symptoms were assessed 3–5 times 10–21 days post inoculation (dpi) and the area under the disease progress curve (AUDPC) calculated. Disease levels were measured on 5–10 plants of each barley line in four independent inoculation experiments.

### *Fusarium culmorum* inoculation

Detached leaves were inoculated with two 5 µl droplets of 10^6^ conidia ml^-1^ of *F. culmorum* isolate Fu42 supplemented with 75 µM deoxynivalenol (DON) as previously described (Chen *et al.*, 2009). Disease spread was photographed and assessed 48h post inoculation by measuring lesion area on leaves using ImageJ (Abramoff *et al.*, 2004). Two independent replicate experiments each containing a minimum of six replicate leaves of each line were inoculated.

### *Oculimacula yallundae* inoculation

The stem bases of 21-day-old plants were inoculated with *O. yallundae* isolate P149 using the method of Chapman *et al.* (2008). The experiment consisting of five replicate blocks each containing five plants of each line was conducted in a randomized block design. Disease assessments were made 8 weeks post inoculation using the scale of Scott (1971) to represent the number of leaf sheaths penetrated and colonized by the fungus.

### qPCR detection of *Ramularia collo-cygni* DNA

Genomic DNA was extracted from prophyll leaves of each mutant and its mother line 21 dpi using the DNeasy Plant DNA extraction kit (Qiagen, Hilden, Germany) to assess *R*. *collo*-*cygni* DNA levels using qPCR (Taylor *et al.*, 2010). A minimum of two leaves from each barley line were sampled from three independent *R*. *collo*-*cygni* inoculation experiments.

### Transcript expression analysis

Transcript levels were assessed in uninfected prophyll leaves sampled from three individual 14-day-old (growth stage 12; Zadoks *et al.*, 1974) plants grown in three separate experiments. RNA was extracted, processed and converted to cDNA as previously described (Colebrook *et al.*, 2012). Levels of gene expression were analysed using quantitative reverse transcription PCR (qRT-PCR) and the Sybr Green Jump Start^TM^ Taq (Sigma) system following the manufacturer’s instructions. PCR amplification and melt curve analysis were performed using a DNA engine Opticon2 Continuous Fluorescence Detector (MJ Research Inc., Alameda, CA, USA) as previously detailed (Colebrook *et al.*, 2012). Five reference genes (elongation factor 1 α, cyclophilin, glyceraldehyde-3-phosphate dehydrogenase, α-tubulin and ubiquitin; McGrann *et al.*, 2009; Colebrook *et al.*, 2012) were used for cDNA normalization (Vandesompele *et al.*, 2002). Transcript abundance was measured using gene specific primers (Supplementary Table S1; Shagimardanova *et al.*, 2010) and expression calculated relative to Steptoe.

### SPAD meter readings for dark-induced senescence

Prophyll leaves from six individual plants at growth stage 12 (Zadoks *et al.*, 1974) were removed and chlorophyll measurements taken from the excised leaves (day 0) using a Chlorophyll Meter SPAD-502 (Konica Minolta, Warrington, UK). Each measurement was produced from the mean of three readings taken from the tip, middle and bottom sections of each leaf. Leaves were then transferred to damp tissue paper in square plastic Petri dishes, wrapped in aluminium foil and placed in a box at room temperature to stimulate dark-induced senescence. Relative chlorophyll measurements were taken at 2, 4 and 6 d after dark treatment as described above. Data were collected from three independent experiments.

### Measuring lesion mimic mutant spots

Plants were sown in F2 compost in 3×3×5cm (P60) and grown in an outside glasshouse under natural light with temperatures ranging from 6°C to 27°C. Prophyll leaves from growth stage 12 (Zadoks *et al.*, 1974) were sampled from each line, photographed and the area of each leaf covered with lesion mimics assessed using ImageJ software (Abramoff *et al.*, 2004). Leaves were collected from plants grown on seven separate occasions.

### Data analysis

All data was analysed using GenStat v. 15 (Payne *et al.*, 2009). *R*. *collo*-*cygni* pathology data measured as the AUDPC and expressed as a percentage of the maximum possible AUDPC was LOGIT transformed and analysed using general linear modelling (GLM) as previously described (McGrann *et al.*, 2014). *F*. *culmorum* data was LOG transformed and analysed using a generalized linear model. The model used to analyse the *R*. *collo*-*cygni* and *F*. *culmorum* pathology data was Experiment+Line. Raw data from the *O*. *yallundae* experiments were analysed with a GLM with block and line as factors. The model used was Block+Line. Variation in *R*. *collo*-*cygni* Log10 DNA levels was assessed using a GLM with experiment and line as factors. The model was Experiment+Line. Dark-induced senescence data was analysed with linear mixed modelling of repeated measurements using the uniform correlation/split plot in time covariance matrix. The fixed model was Experiment*Day*Line and the random model was Leaf*Day. Leaf lesion area variation of *nec1* and *mlo5* mutants was analysed with a GLM with experiment and line as factors. The model used was Experiment+Line. Significant differences between lines, and between lines and days in the dark-induced senescence experiment, were subsequently assessed using a t-test conducted within the specific model for each analysis performed.

## Results

### Development of Ramularia leaf spot symptoms on barley lesion mimic mutants

Lesion mimic phenotypes of the *nec* mutant lines have been reported elsewhere (Rostoks *et al.*, 2003; Keisa *et al.*, 2011) and range from leaves expressing a few small necrotic spots (*nec1*), to leaves displaying numerous necrotic patches covering a large proportion of the leaf area (*nec8*) and plants with leaves that show a chlorotic phenotype with a few necrotic regions (*nec9*; Supplementary Fig. S1). Ramularia leaf spots were distinguished based on the characteristic reddish brown colour and ‘box-shape’ of the lesions, which are delineated by the leaf veins. This distinctive appearance of the RLS lesions contrasts with the spotting and necrosis associated with the different lesion mimic phenotypes which varied in colour from dark brown to black, dependent on the mutant line, and were typically spread across leaf veins and so allowed accurate scoring of disease symptoms on each mutant.

**Fig. 1.**
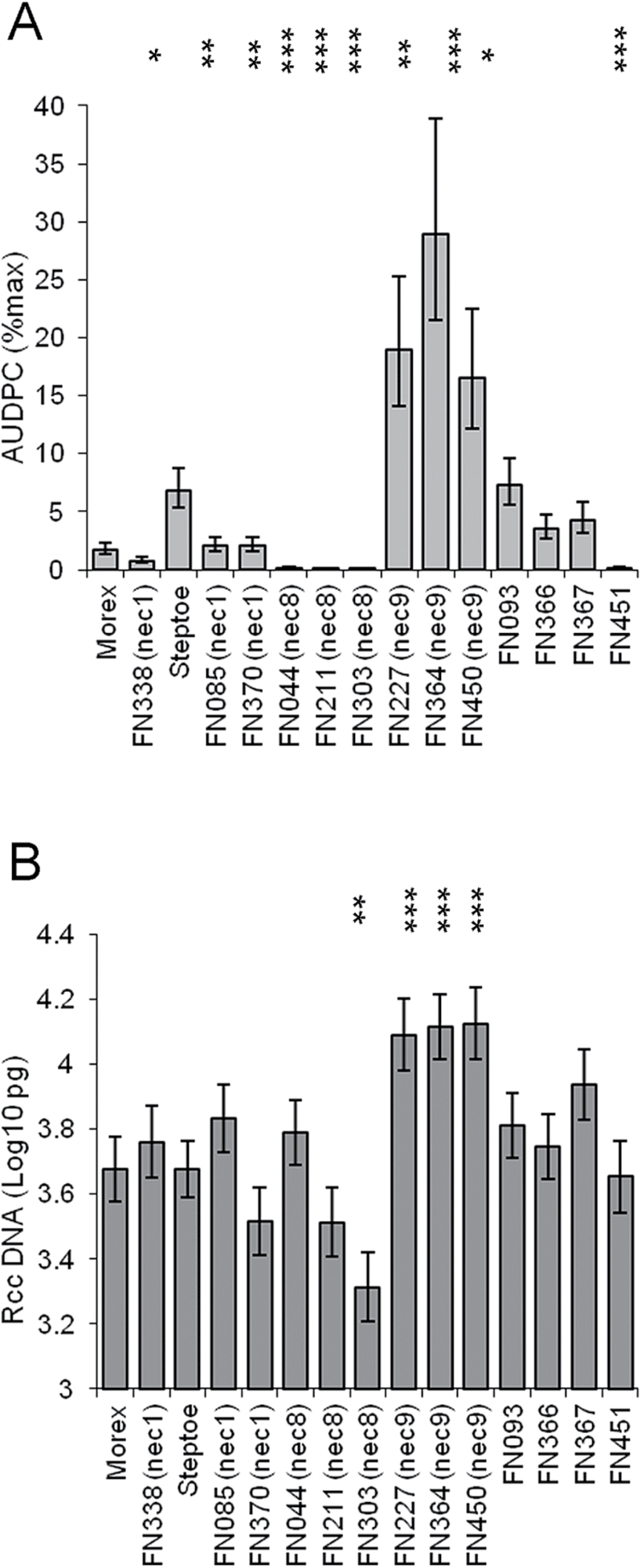
Development of Ramularia leaf spot (RLS) in barley lesion mimic mutants in cv. Steptoe or cv. Morex. (A) Disease symptom expression presented as the area under disease progress curve of RLS (B) *R*. *collo*-*cygni* DNA in leaves of lesion mimic mutants. ^***^ *P* <0.001; ^**^ *P* <0.01; *P <0.05.

**Fig. 2.**
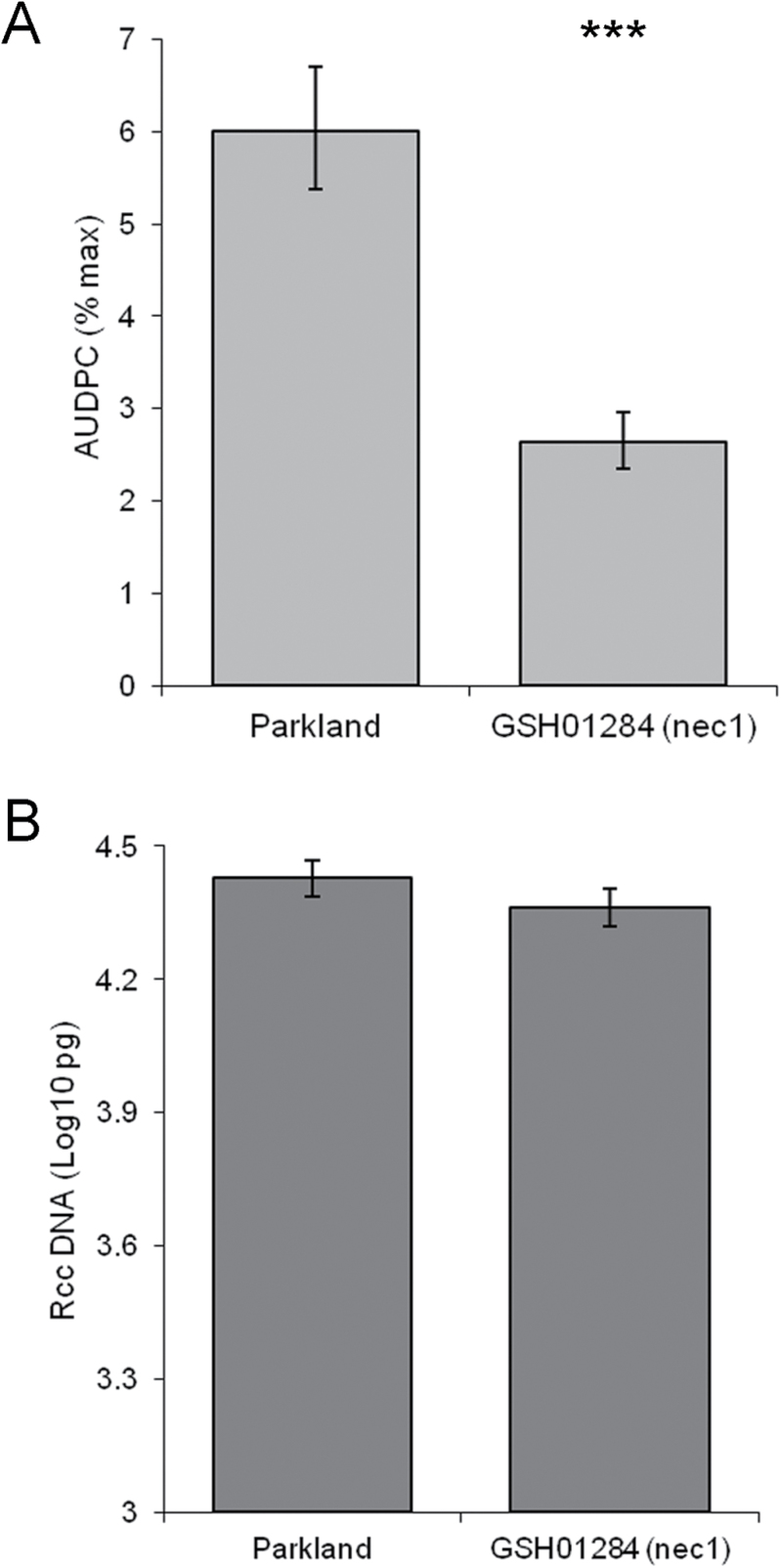
Development of Ramularia leaf spot (RLS) in barley lesion mimic mutants in cv. Parkland. (A) Disease symptom expression presented as the area under disease progress curve of RLS (B) *R*. *collo*-*cygni* DNA in leaves of lesion mimic mutants. ^***^ *P* <0.001; ^**^ *P* <0.01; *P <0.05.

Mutation of the *NEC1* locus significantly decreased the development of Ramularia leaf spots in FN085 and FN370 (*P<*0.01) compared to Steptoe, and disease progressed more slowly in the *nec1* mutant FN338 than in the Morex parent line (*P*=0.05; Fig. 1A). In a separate set of experiments the *nec1* mutant GSH01284 also showed reduced Ramularia leaf spot development (*P*<0.001) compared to its parent line Parkland (Fig. 2A). All three *nec8* mutants, FN044, FN211 and FN303, exhibited significantly fewer Ramularia leaf spot symptoms than Steptoe (*P*<0.001) as did the mutant FN451 (*P*<0.001; Fig. 1A). In contrast the three *nec9* mutants exhibited significantly more disease symptoms than Steptoe (FN227 *P<*0.01, FN364 *P*<0.001, FN450 *P<*0.05). Representative images of RLS symptoms on Steptoe and lesion mimic phenotype lines in this background are shown for *nec1* (FN085), *nec8* (FN303) and *nec9* (FN227, FN364, FN450) plants in Supplementary Fig. S1. None of the mutants FN093, FN366 or FN367 showed significantly different levels of Ramularia leaf spot development compared to Steptoe wild-type (Fig. 1A).

### Quantification of *R. collo-cygni in planta* using fungal DNA levels

None of the mutants FN044, FN085, FN093, FN211, FN338, FN366, FN367, FN370, FN451 or GSH01284 had levels of *R*. *collo*-*cygni* DNA significantly different to their respective mother lines (Figs 1B, 2B) but the *nec8* mutant FN303 had significantly less fungal DNA than Steptoe (*P<*0.01; Fig. 1B). All three *nec9* mutants had significantly increased levels of fungal DNA compared to Steptoe (FN227 *P<*0.01, FN364 *P*=0.001, FN450 *P<*0.01; Fig. 1B), consistent with the observed increase in disease symptom development in these mutants.

### Effect of *nec* mutants on symptom development of *O. yallundae* and *Fusarium culmorum*

**Fig. 3.**
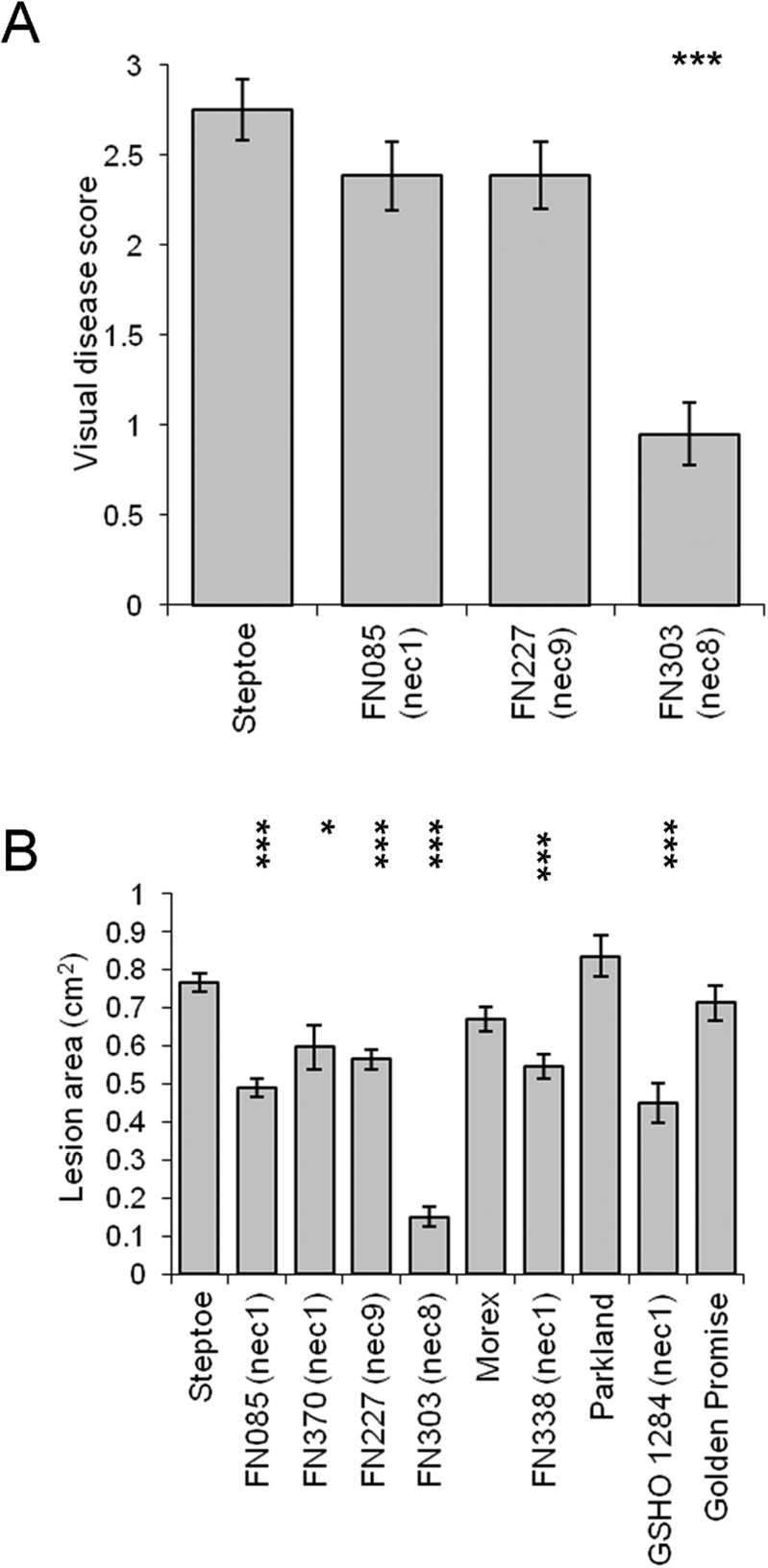
Development of disease symptoms caused by (A) *Oculimacula yallundae*, (B) *Fusarium culmorum* on selected barley lesion mimic mutants. ^***^ *P* <0.001; ^**^ *P* <0.01; *P <0.05.

The response of Steptoe and the FN085 (*nec1*), FN303 (*nec8*) and FN227 (*nec9*) mutants was also tested against the facultative fungal pathogens *O. yallundae* and *F. culmorum*. No significant differences in *O. yallundae* disease scores were observed for FN085 (*nec1*) or FN227 (*nec9*) compared to Steptoe, but disease development was significantly reduced on FN303 (*nec8*) (*P*<0.001; Fig. 3A). The size of *F*. *culmorum* lesions were significantly reduced on the leaves of all three mutants compared to those on the parent line (*P*<0.001). The effect of the *nec1* mutation on *F*. *culmorum* lesion size was confirmed by screening additional *nec1* mutants in the Steptoe (FN370), Morex (FN338) and Parkland (GSH01284) backgrounds. Presence of the *nec1* mutation reduced *F*. *culmorum* lesion size in Morex (*P<*0.01; Fig. 3B) and Parkland (*P*<0.001, Fig. 3B) and in the Steptoe *nec1* mutant FN370 (*P*<0.05; Fig. 3B).

### qRT-PCR transcript profiling antioxidant and defence-related genes in selected barley lesion mimic mutants

During necrotrophic development facultative fungi can exploit elevated *in planta* ROS production to promote colonization (Heller and Tudzynski, 2011). Therefore the expression levels of ROS scavenging transcripts were measured in *nec1* (FN085), *nec8* (FN303), and *nec9* mutants (FN227, FN364, FN450) to assess the constitutive antioxidant status of these plants in pathogen-free conditions. All three allelic *nec9* mutants were investigated to test for common changes in the antioxidant system potentially related to enhanced susceptibility to RLS. Changes in expression of genes involved in antioxidant activity were generally low in the mutants; none of the mutants consistently exhibited a greater than 2-fold increase in transcript levels of any of the antioxidant genes tested (Table 2). All five mutants showed greater than 2-fold decrease in *Glutathione peroxidase 2* (GPX2) transcript levels but this repression was strongest in the three *nec9* mutants. Expression of *Glutathione reductase 1* (GR1) was also reduced in all five mutants but transcript repression greater than 2-fold was only recorded for the three *nec9* mutants (FN227, FN364, FN450). There was no noteworthy change in expression of *Glutathione peroxidase 1* (GPX1), *Copper-zinc superoxide dismutase 1* (CSD1) or *Catalase 1* (Cat1) in any of the mutants compared to Steptoe whereas as *Catalase 2* (Cat2) showed more than a 2-fold decrease in transcript levels in FN450 alone. Transcript levels of *Ascorbate peroxidase 1* (APX1) and *Ascorbate peroxidase 2* (APX2) were reduced in FN085, FN303, FN364 and FN450 but not FN227 (Table 2).

**Table 2.** qRT-PCR expression analysis of barley antioxidant and defence-related transcripts in selected lesion mimic mutants in cv. Steptoe

	Steptoe	FN085 (*nec1* )	FN303 (*nec8* )	FN227 (*nec9* )	FN364 (*nec9* )	FN450 (*nec9* )
**Antioxidants**						
Ascorbate peroxidase 1 (APX1)	1.000 (±0.129)	0.486 (±0.104)	*0.405* (±0.075)	0.802 (±0.174)	0.584 (±0.084)	0.544 (±0.093)
Ascorbate peroxidase 2 (APX2)	1.000 (±0.245)	0.543 (±0.123)	0.656 (±0.110)	1.270 (±0.588)	0.580 (±0.147)	*0.398* (±0.077)
Catalase 1 (CAT1)	1.000 (±0.348)	1.369 (±0.518)	1.745 (±0.516)	1.208 (±0.427)	1.598 (±0.505)	1.901 (±0.392)
Catalase 2 (CAT2)	1.000 (±0.386)	1.906 (±0.693)	1.960 (±0.402)	1.156 (±0.342)	1.428 (±0.467)	*0.403* (±0.079)
Glutathionine peroxidase 1 (GPX1)	1.000 (±0.320)	0.941 (±0.280)	1.240 (±0.210)	1.208 (±0.252)	1.427 (±0.285)	1.463 (±0.346)
Glutathionine peroxidase 2 (GPX2)	1.000 (±0.126)	*0.498* (0.123)	*0.370* (±0.068)	*0.244* (±0.056)	*0.229* (±0.048)	*0.066* (±0.008)
Copper/Zinc superoxide dismutase 1 (CSD1)	1.000 (±0.125)	1.595 (±0.455)	1.38 (±0.221)	1.05 (±0.088)	1.432 (±0.246)	1.269 (±0.198)
Glutathione reductase 1 (GR1)	1.000 (±0.058)	0.524 (±0.145)	0.672 (±0.142)	*0.471* (±0.064)	*0.434* (±0.076)	*0.420* (±0.060)
**Defence-related**						
Pathogenesis-related 1 (PR1)	1.000 (±0.245)	**125.217** (±35.130)	**94.328** (±15.461)	**51.110** (±13.154)	**53.724** (±23.629)	**39.386** (±7.111)
BAX-inhibitor 1 (BI-1)	1.000 (±0.379)	1.549 (±0.429)	**3.011** (±0.480)	0.881 (±0.194)	0.925 (±0.199)	0.571 (±0.089)

Mean normalised expression values are rescaled relative to Steptoe to show fold-change differences in transcript levels in mutant leaves compared to wild-type: >2-fold repressed, italics; >2-fold induced, bold (standard error shown in parentheses).

Transcript levels of the defence-related *Pathogenesis-related 1* (*PR1*) and the cell death regulator *Bax-1 inhibitor* (*BI-1*) were also monitored in all five lesion mimic mutants. *PR1* transcript levels were increased more than 30-fold in all five lesion mimic mutants tested (Table 2), whereas the *BI-1* transcript was induced (~3-fold) in the *nec8* mutant only (Table 2).

### Analysis of dark-induced senescence in barley lesion mimic mutants

**Fig. 4.**
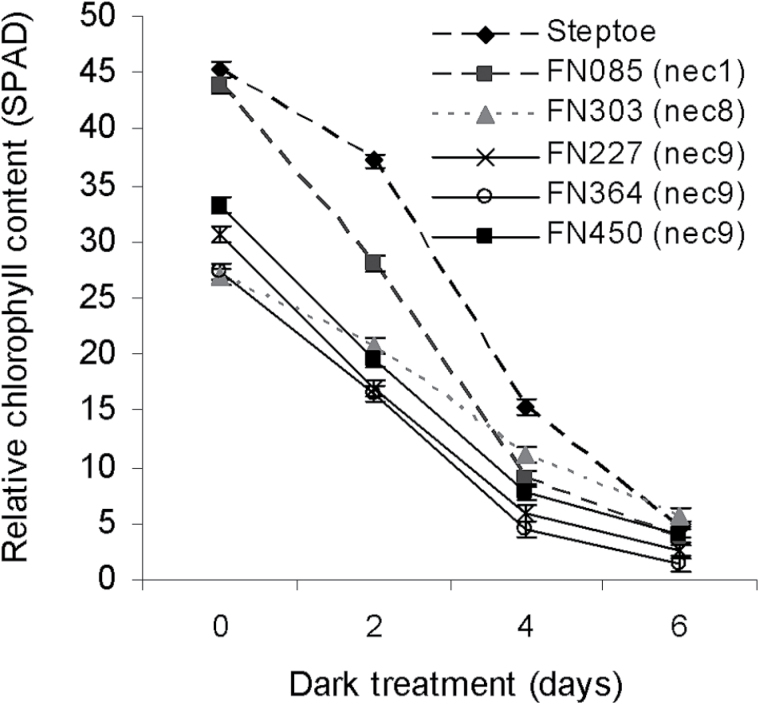
Rate of dark-induced senescence in selected lesion mimic mutants.

Senescence is a cell death process that is generally believed to assist with maintaining plant health by nutrient remobilization to areas of new vegetative growth (Jones, 2001). Lesion mimic mutants often also exhibit accelerated senescence (Lorrain *et al.*, 2003; Moeder & Yoshioka, 2008). Dark-induced senescence was used to determine if *nec1*, *nec8* and *nec9* mutations affect leaf senescence. Leaves of the *nec8* and *nec9* mutants had significantly lower SPAD readings than the parent Steptoe line at the time of detachment from the plant and before senescence was induced (*P*<0.001). The *nec1* mutant, however, had SPAD readings comparable to those of Steptoe (Fig. 4). After two and four days of dark treatment the *nec1*, *nec8* and *nec9* mutants all had significantly lower (*P*<0.001) SPAD readings than wild-type. After six days of dark treatment all lines had much reduced SPAD readings and no significant differences were observed between Steptoe and the *nec1* and *nec8* lines and the *nec9* mutant FN450. Two *nec9* mutants however (FN227 and FN364) had senesced to a greater degree than Steptoe and had significantly lower SPAD readings than the wild-type (*P*<0.05 and *P*<0.01 respectively).

### Functional relationship between NEC1 and MLO

**Fig. 5.**
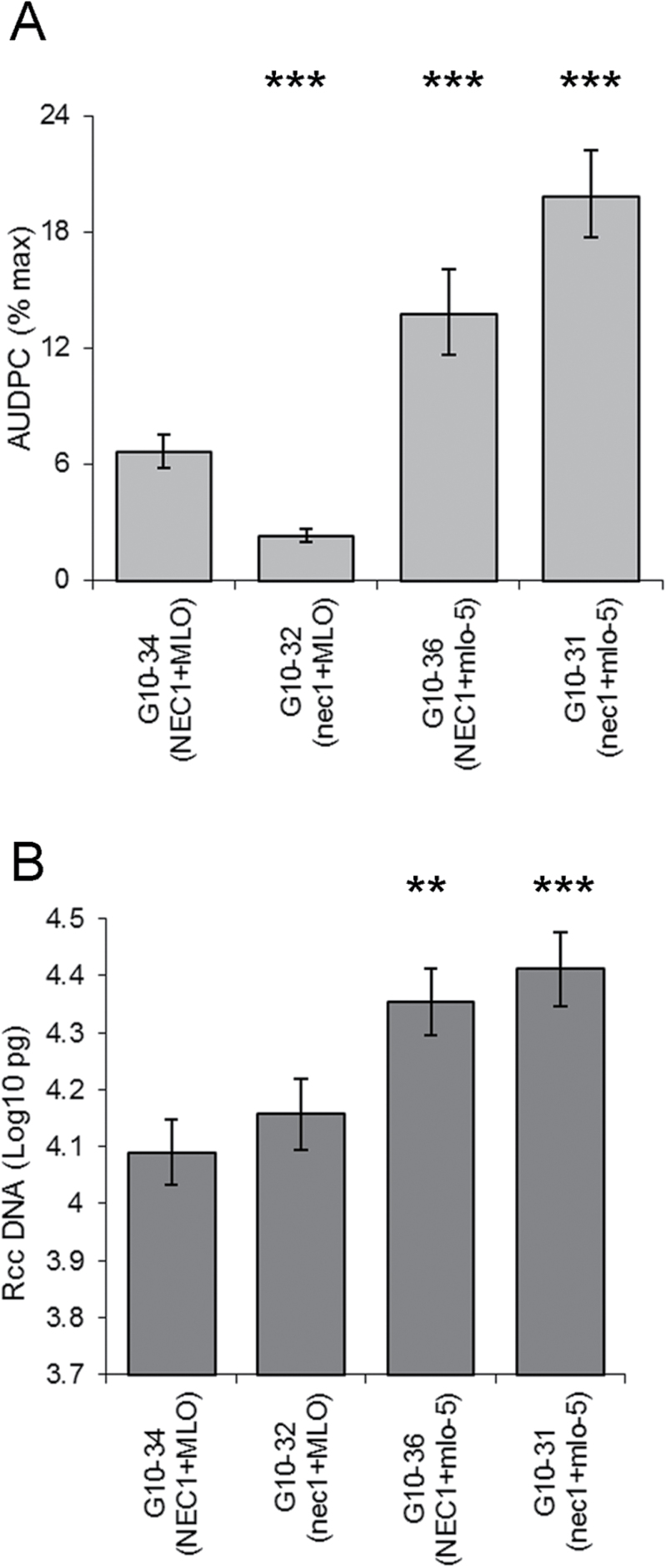
Effect of the *mlo*-*5* mutation on Ramularia leaf spot (RLS) development in *nec1* mutant plants. (A) Disease symptom expression presented as the area under disease progress curve of RLS (B) *R*. *collo*-*cygni* DNA in leaves of lesion mimic mutants. ^***^ *P* <0.001; ^**^ *P* <0.01.

As both *NEC1* and *MLO* have been implicated in regulation of cell death processes (Peterhänsel *et al.*, 1997; Piffanelli *et al.*, 2002; Ma and Berkowitz, 2011; Moeder *et al.*, 2011) the relationship between the two genes was tested using a *nec1*+*mlo-5* double mutant (Keisa *et al.*, 2011). Firstly whether the *nec1* mutation affected *mlo*-*5*-enhanced RLS susceptibility was examined. The *nec1* single mutant (G10-32) showed reduced RLS disease symptom development (*P*<0.001; Fig. 5A) compared to the *NEC1*+*MLO* wild-type line (G10-34) whereas the *mlo-5* mutant line (G10-36) had increased disease levels compared to the wild-type (*P*=0.001; Fig. 5A) as previously observed (Figs 1A, 2A). The *nec1*+*mlo-5* double mutant (G10-31) had significantly increased disease development compared to the *nec1* single mutant and the wild-type lines (*P*=0.001; Fig. 5A). *R*. *collo*-*cygni* DNA levels were also higher in both the *mlo-5* single mutant and *nec1*+*mlo-5* double mutant compared to *nec1* single mutant and the wild-type lines (Fig. 5B). There was no significant difference in fungal DNA levels between the *nec1* mutant and wild-type line (*P*=0.435; Fig. 5B).

**Fig. 6.**
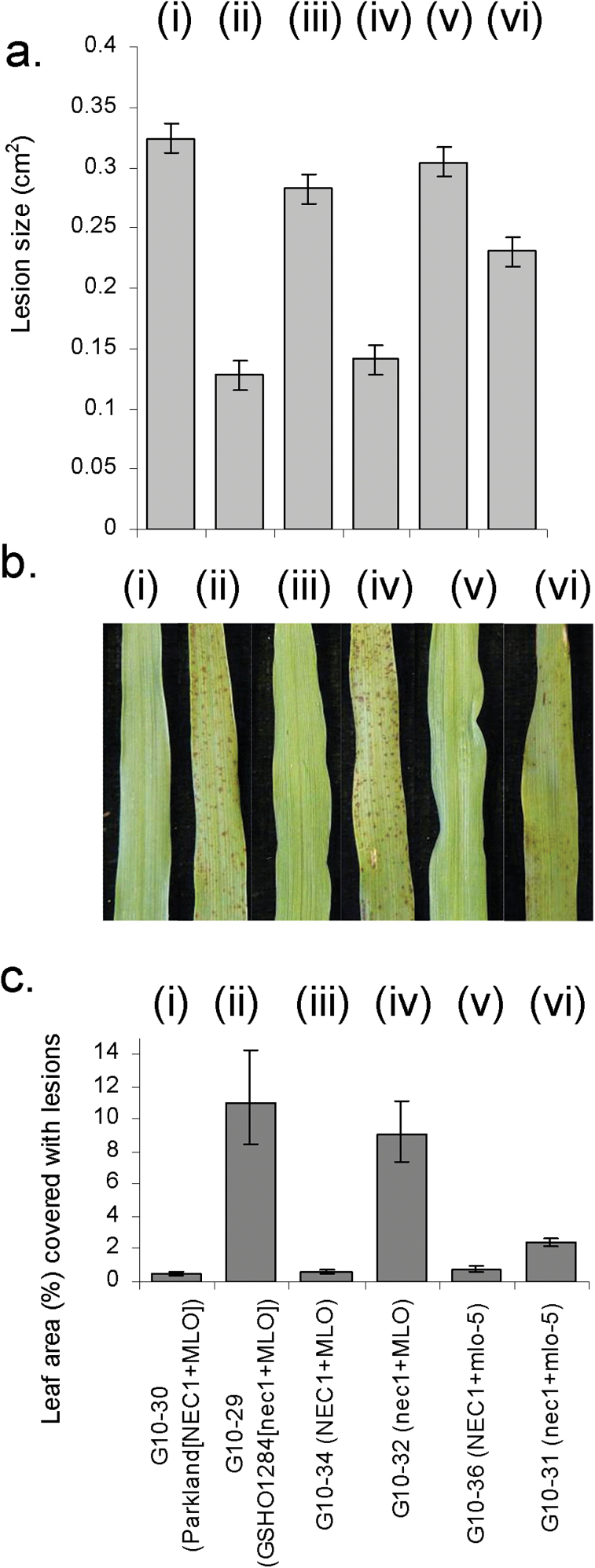
Effect of the *mlo*-*5* mutation on *Fusarium culmorum* lesion formation and lesion mimic development in *nec1* mutant plants. (A) *F*. *culmorum* development was assessed by measuring the diameter of the lesion on the leaf. (B) Images of lesion mimic development on (i) *NEC1*+*MLO* [G10-30/Parkland; parent line], (ii) *nec1*+*MLO* [G10-29/ GSHO1284], (iii) *NEC1*+*MLO* [G10-34], (iv) *nec1*+*MLO* [G10-32], (v) *NEC1*+*mlo*-*5* [G10-36], (vi) *nec1+mlo*-*5* [G10-31]. (C) Lesion mimic development in each of the different lines in (B) was assessed as a proportion of the leaf area covered with necrotic spots using ImageJ software.

**Fig. 7.**
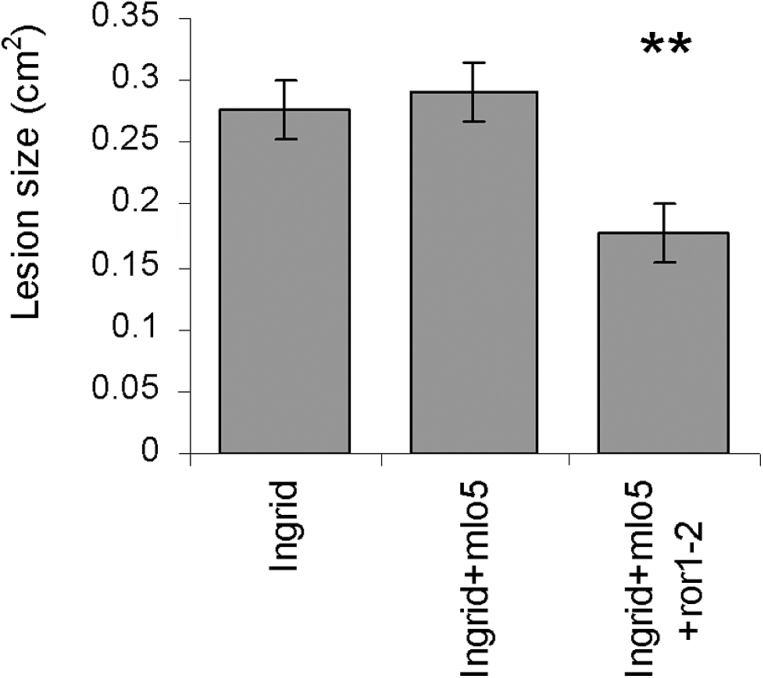
Effect of *ror1-2* mutation on *Fusarium culmorum* lesion formation. *F*. *culmorum* development was assessed by measuring the diameter of the lesion on the leaf. ^**^ *P* <0.01.

The *mlo*-*5* mutation has been implicated in increasing susceptibility to *Fusarium* infection of barley. This mutation was shown to result in increased *F*. *graminearum* development in barley caryopses (Jansen *et al.*, 2005). Therefore the effect of the *mlo-5* mutation alone and *mlo-5* and *nec1* together on the *F*. *culmorum* lesion development was examined in foliar assays. In the detached leaf assays used in this study, none of the different *mlo* mutations in various genetic backgrounds showed a significant effect on *F*. *culmorum* lesion development (Supplementary Fig. S2). As observed above, lesions were smaller in the presence of the *nec1* mutation (Fig. 6A). However, *F*. *culmorum* lesions were significantly larger in the *nec1*+*mlo-5* double mutant than in the *nec1* single mutant (G10-32; *P*<0.001). Functional *ROR* genes are required for susceptibility to the necrotrophic stage of *R*. *collo*-*cygni* in *mlo* plants (McGrann *et al.*, 2014). The effect of the *ror1-2* mutation of on *F*. *culmorum* lesion development was assessed using the detached leaf assay. The presence of the *ror1-2* mutation in the *mlo-5* background significantly reduced lesion size compared to either the single *mlo-5* or wild-type Ingrid plants (Fig. 7; *P*<0.01).

The relationship between *MLO* and *NEC1* was further examined by visual assessment of the *nec1* lesion mimic phenotype itself in the different genotypes. As expected both *nec1* single mutants (G10-32 and GSH01284) had significantly more leaf area covered with lesions than their respective *NEC1*+*MLO* wild-type lines (G10-34, Parkland) (Fig. 6B, C; *P*<0.001). The *nec1*+*mlo-5* double mutant showed increased lesion coverage compared to *NEC1*+*MLO* line (G10-34; *P*<0.001) but had significantly less lesion area than the *nec1*+*MLO* mutant (G10-32; Fig. 6C; *P*<0.001). There was no significant difference in lesion coverage between *NEC1*+*MLO* and *mlo-5* single mutant (G10-35; *P*=0.422).

## Discussion

Programmed cell death is critical for many plant developmental processes and defence responses (Jones, 2001). Cell death is typically a component of the defence response against biotrophic pathogens, whereas its role in interactions between plants and facultative pathogens with hemibiotrophic or necrotrophic lifestyles can be somewhat ambivalent (Jarosch *et al.*, 1999; Kumar *et al.*, 2001; Mengiste, 2012; Saville *et al.*, 2012; Wright *et al.*, 2013). This investigation, using a collection of diverse lesion mimic mutants that differentially regulate disease symptom expression caused by facultative fungi on barley, highlights the complex relationship between host cell death and disease development in plants.

Reduced disease development by all three facultative pathogens was observed on each of the three *nec8* alleles tested. *nec8* mutation also confers enhanced resistance against the biotroph *Puccinia graminis* and is thought to encode a cation/proton exchange protein, HvCAX1 (Zhang *et al.*, 2009). Regulation of ion flux is critical to maintain cellular homeostasis for plant growth and development as well as for directing plant responses to pathogens (Dangl *et al.*, 1996). Compared to the other lesion mimic mutants tested the *nec8* plants display the most severe necrotic symptoms and growth defects. The growth defects of *nec8* mutants together with increased transcript expression of HR and defence-related genes (Rostoks *et al.*, 2003; Zhang *et al.*, 2009; Table 2) suggests that *nec8* may be a constitutively activated defence mutation.

*nec9* mutations had contrasting effects on symptom expression caused by each fungus, showing no effect on *O*. *yallundae* development, reducing the size of *F*. *culmorum* lesions but increasing RLS symptom expression (Figs 1A, 3). The lesion mimic phenotype of *nec9* mutants resulted in more chlorotic leaves with fewer necrotic spots than either the *nec1* or *nec8* lines (Supplementary Fig. S1). No obvious symptom on the stem was observed which might be why no effect on development of *O*. *yallundae*, a stem-base pathogen, was detected. Leaves of *nec9* plants had low relative chlorophyll measurements compared to wild-type, as expected given their chlorotic phenotype (Fig. 4). Nevertheless, they did not exhibit a reduced rate of dark-induced senescence compared to the Steptoe parent line. Leaf senescence has been implicated in the pathology of both *Fusarium* spp. and *R*. *collo*-*cygni* and in both cases accelerated senescence is associated with promotion of disease (Schützendübel *et al.*, 2008; Chen *et al.*, 2009). Therefore it is unlikely that altered senescence is responsible for the difference in symptom development caused by these two pathogens in *nec9* mutants.

The *nec1* mutations reduced symptom development caused by *R*. *collo*-*cygni* (Figs 1A, 2A, 5A) and *F*. *culmorum* (Figs 3B, 6A) but not by the stem base infecting *O*. *yallundae* (Fig. 3A)*. NEC1* is predicted to encode a cyclic nucleotide-gated ion channel (CNGC) 4 protein (Rostok *et al*., 2006) based on sequence similarity with its *A*. *thaliana* orthologue, *HLM1*, which has been proposed as an essential signalling component common to both the HR and disease resistance responses (Balague *et al.*, 2003). The reduction in disease development caused by *F*. *culmorum* and *R*. *collo*-*cygni* may be associated with the accelerated onset of cell death in *nec1* mutants. *R*. *collo*-*cygni* has a prolonged endophytic phase before becoming necrotrophic although the cues for this transition are currently not understood (Walters *et al.*, 2008). Prevention of cell death in DELLA gain-of-function (GOF) barley mutants resulted in enhanced RLS symptom formation whereas disease levels were significantly reduced in DELLA loss-of-function (LOF) mutants that exhibit enhanced cell death. This suggests that RLS symptom development is reduced when the host has an enhanced propensity to initiate cell death (Saville *et al.*, 2012) as is observed here in the *nec1* mutant (Fig. 6B, C).

**Fig 8.**
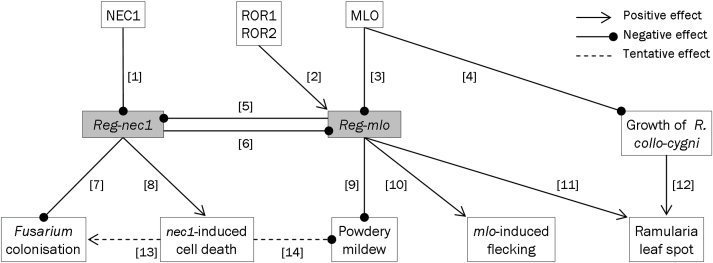
A hypothesis for the joint effect of *NEC1* and *MLO* on various pathogenic fungi and cell death. The model is based on the following data: *nec1*-dependent cell death (CD) [1, 8] Fig. 6B, C; effect of *nec1* on *Fusarium* colonisation [7] Fig. 6a; effect of *mlo-5* on *nec1*-dependent traits [3, 5] Fig. 6; *mlo*-dependent stimulation of *Ramularia collo-cygni* (*Rcc*) fungus [4] and Ramularia leaf spot (RLS) symptoms [3, 11] Fig. 5, McGrann *et al.* (2014), Brown & Makepeace (2009); suppression of RLS [6, 11] but not *Rcc* growth [12] in *nec1+MLO* plants, Fig. 5; *mlo*-dependent mildew resistance [3, 9] Jørgensen (1992), Büschges *et al*. (1997); *mlo*-dependent CD [3, 10] Peterhänsel *et al*. (1997). Traits enhanced by *ROR* genes [2] are mildew resistance [10] (Freialdenhoven *et al.*, 1996; Collins *et al.*, 2003), *mlo*-dependent CD [11] (Peterhänsel *et al.*, 1997), RLS [12] (McGrann *et al.*, 2014), and *Fusarium* colonisation (Fig. 7; *ROR1* tested but not *ROR2*). Growth of *Rcc* is not affected by *ROR* genes (McGrann *et al.*, 2014). The Reg-nec1 and Reg-mlo components are hypothetical, as are the effects of *nec1*-dependent CD on *Fusarium* [13] (Fig. 6) and mildew [14] (Keisa *et al.*, 2011). See Discussion and Supplementary Fig. S3 for detailed description of the model.

Although the *nec1* mutants exhibited reduced RLS symptoms (Fig. 5A) there was no effect on fungal biomass (Fig. 5B) suggesting that *NEC1* may be involved in the regulation of the transition from endophyte to necrotroph rather than restricting growth of the *R*. *collo*-*cygni* fungus (see Fig. 8). *F*. *culmorum* is a hemibiotroph that has a short biotrophic phase (Scherm *et al.*, 2013) that is likely to occur during colonization of the cereal ear tissue as observed for the related species *F*. *graminearum* (Brown *et al.*, 2010). To date there is no evidence to suggest there is a biotrophic phase during foliar infections. Wounding the leaf during the inoculation process may enable the fungus to begin necrotrophic development immediately. If a biotrophic phase does occur during *F*. *culmorum* leaf infection it is conceivable that the mechanisms that result in lesion mimic formation also function in resistance towards this stage of pathogen development. However, the DELLA GOF and LOF mutants showed opposite effects on *F*. *graminearum* symptom development compared to *R*. *collo*-*cygni* (Saville *et al.*, 2012) indicating that enhanced cell death promotes *Fusarium* symptoms. The negative effect of *nec1* on both RLS and *Fusarium* symptom development compared to the contrasting effects of DELLA (Saville *et al.*, 2012) and *mlo* mutations (Supplementary Fig. S2) on these diseases suggests that specific pathogens may be affected differentially by distinct cell death pathways (discussed below). Alternatively *NEC1* may regulate other pathways that confer disease resistance. The *nec1* mutation increases endogenous auxin levels whilst enhancing stomatal closure (Keisa *et al.*, 2013). Application of auxin can prime cereal plants for resistance against *F*. *culmorum* (Petti *et al.*, 2012) whereas airborne *R*. *collo*-*cygni* spores germinate on leaves and penetrate through stomata (Stabentheiner *et al.*, 2009). These alternative effects of *nec1* on barley may explain why these mutants are resistant to *F*. *culmorum* and *R*. *collo*-*cygni* independent of cell death.

*NEC1* has a complex interaction with the *MLO* gene. Fig. 8 presents a hypothesis to account for the phenotypes controlled by these genes, including the data in Figs 5, 6 and 7 as well as previously published data. Together, these data indicate that rather than operating through a linear relationship, the interaction between *MLO* and *NEC1* is a dynamic two-way process with outcomes that are specific to the particular pathogen. There may be a central role for a process by which the regulatory mechanisms controlled by NEC1 and MLO suppress each other under normal conditions.

Comparing plants with the *NEC1*, *MLO* and *nec1 MLO* genotypes, the *nec1* mutation increases cell death (Fig. 6B, C; [1, 8] in Fig. 8) and reduces colonization by *F. culmorum* (Fig. 6a; [7]). Whether *nec1*-dependent cell death contributes to suppressing *F. culmorum* lesions [13] or the two traits are separate effects is not yet known, so they are shown as possibly independent in Fig. 8.

Comparing *NEC1 MLO* and *NEC1 mlo-5* plants, *mlo-5* increased both the growth of *R. collo-cygni* (Fig. 5B; [4]) and expression of RLS symptoms (Fig. 5A; [12]), as in previous work (Brown & Makepeace, 2009; McGrann *et al.*, 2014). Development of RLS symptoms does not depend solely on infection by *R. collo-cygni* and other factors determine whether or not the disease appears in infected leaves (McGrann *et al.*, 2014; [3, 11]). In contrast to their effects on RLS, loss-of-function *mlo* alleles confer strong resistance to powdery mildew (Jørgensen, 1992; Büschges *et al.*, 1997; [3, 9]). Spontaneous cell death and necrotic flecks can appear in *mlo* plants (Wolter *et al.*, 1993; Büschges *et al.*, 1997; [3, 10]) but few such lesions appeared in our experiments and there was no significant difference between amounts of flecking on *mlo-5* and *MLO* plants (Fig. 6B, C; also unpublished observations on near-isogenic lines bred from cvv. Ingrid, Pallas, Haisa and Malteria Heda). Hence control of the necrotic flecking pathway in *mlo-5* plants is at least partly separate from the *nec1*-dependent lesion-mimic pathway. It is sensitive to environmental conditions and can be associated with mildew-resistance and RLS-susceptibility but is not required for these disease phenotypes. We propose that there may be a common regulatory mechanism, marked ‘Reg-mlo’ in Fig. 8. This is repressed by wild-type *MLO* [3], suppresses mildew [9], promotes RLS symptoms [11] and enhances cell death in certain environments [10].

The *Fusarium*-susceptible phenotype without necrotic lesions in *NEC1* plants is independent of the plant’s allele at the *MLO* locus (Fig. 6; Supplementary Fig. S2). In *nec1* plants, by contrast, functional MLO is required for full expression of both cell death [8] and *Fusarium*-resistance [7] (Fig. 6; [3, 5]). As *nec1* is a loss-of-function mutation, MLO presumably interacts with a hypothetical regulator, labelled ‘Reg-nec1’ in Fig. 8, which is strongly repressed by NEC1 [1] and is therefore stimulated in the *nec1* genotype, rather than by NEC1 itself. The reason for proposing that MLO influences the *nec1*-dependent pathway indirectly via an interaction between ‘Reg-mlo’ and ‘Reg-nec1’ is based on the effect of the *ror1-2* mutation on *Fusarium* colonization (see below).

The effect of *nec1* on RLS is complex because it depends on the allele at the *MLO* locus. It was associated with reduced RLS symptoms in *MLO* plants but with increased RLS in *mlo-5* hosts (Fig. 5A) although it did not alter amounts of *R. collo-cygni* in the leaf significantly in either case (Fig. 5B). This is further evidence that factors in addition to the presence of the pathogen affect the etiology of RLS (McGrann *et al.*, 2014). These contrasting effects may be explained by mutual inhibition of the *nec1*-dependent and *mlo-5*-dependent pathways. In *nec1 MLO* plants, ‘Reg-mlo’ is repressed by MLO [3]. ‘Reg-nec1’ is enhanced by the loss of NEC1 function [1], which further represses ‘Reg-mlo’ [6]. The outcome of the interaction between the pathways is that *nec1 MLO* plants are even less susceptible to RLS [11] than *NEC1 MLO* plants. In *nec1 mlo-5* plants, the absence of MLO protein causes repression of ‘Reg-mlo’ to be lifted [3], so the higher concentration of ‘Reg-mlo’ leads to increased repression of ‘Reg-nec1’ [5]. The lower level of ‘Reg-nec1’ then causes the level of ‘Reg-mlo’ to be higher than in *NEC1 mlo-5* plants, so that *nec1 mlo-5* plants are highly susceptible to RLS [11]. Moreover, the loss of *MLO* stimulates growth of the *R. collo-cygni* fungus [12]. Note that the proposed ‘Reg-nec1’ and ‘Reg-mlo’ entities are hypothetical mechanisms but their existence is required to account for the interacting effects of *NEC1* and *MLO* on diverse traits. They may be, for example, proteins (or sets of proteins) that inhibit one another, signalling pathways that act in opposition to one another, or conflicting physiological states within the leaf tissue.

The *nec1* mutation also reduces susceptibility to mildew in the *MLO* genotype (Keisa *et al.*, 2011). This is not explained by the effect of ‘Reg-nec1’ on ‘Reg-mlo’. Instead, it could result from necrotic lesions that reduce susceptibility to *B. graminis*, an obligate biotroph [14].

The complexity of the links between *NEC1* and *MLO* is further indicated by the role that *ROR* genes play in traits affected by their interaction. *ROR1* and *ROR2* act in opposition to many of the functions of *MLO* although they may not interact with *MLO* directly (Collins *et al.*, 2003). They are required for full expression of several traits: resistance to mildew (Collins *et al.*, 2003), which is particularly striking in *mlo* plants (Freialdenhoven *et al.*, 1996), suppression of *mlo*-dependent cell death (Peterhänsel *et al.*, 1997) and development of RLS (McGrann *et al.*, 2014). They do not, however, significantly alter *R*. *collo*-*cygni* DNA levels in infected leaves (McGrann *et al.*, 2014). The effect of *ror* mutants on RLS but not *R*. *collo*-*cygni* is therefore similar to that of *nec1* (Figs 1, 2, 5), suggesting that both ROR proteins, NEC1 and MLO are all required for full activity of the ‘Reg-mlo’ mechanism. *ROR1* is also required for full expression of susceptibility to *Fusarium* (Fig. 7), which, like *R. collo-cygni*, is a non-biotrophic pathogen. The simplest way in which the ROR proteins could have all the effects observed on both *nec1*-dependent and *mlo*-dependent traits would be if they enhanced the effect of ‘Reg-mlo’, the protein or process which is inhibited by MLO [2]. The effects of the *nec1* and *mlo* mutations, separately and jointly, and of the *ror1-2* mutation combined with *mlo-5* on all six biotic and abiotic syndromes discussed here are summarized in Fig. 8 and Supplementary Fig. S3.

Control of host cell death has been proposed as one of the mechanisms by which compatibility in cereals against biotrophic and facultative fungal pathogens is differentially regulated (Saville *et al.*, 2012). As observed in this study the contribution of cell death to plant defence appears diverse even to fungi with similar lifestyles. Subtle alterations to the signalling cascades that define the cell death programme may result in differences in pathogen perception or defence signalling. Alternatively altered cell death pathways may change or eliminate the specific host cues that are required by particular fungi to trigger switches in life habits leading to a spectrum of responses to facultative pathogens.

## Supplementary data

Supplementary data can be found at *JXB* online.

Supplementary Table S1. qRT-PCR primers used in this study.

Supplementary Fig. S1. Lesion mimic and Ramularia leaf spot infection phenotypes on leaves of Steptoe, *nec1* (FN085), *nec8* (FN303) and *nec9* (FN227, FN364, FN450) mutant plants.

Supplementary Fig. S2. Effects of independent *mlo* mutations in different barley genetic backgrounds on *Fusarium culmorum* lesion development.

Supplementary Fig. S3. A proposed network of interaction between *NEC1* and *MLO* and their effects on several traits.

Supplementary Data
