# Supplementary material for: Differential effects of lesion mimic mutants in barley on disease development by facultative pathogens
Source: J Exp Bot. 2015 Apr 8;66(11):3417–28. doi: 10.1093/jxb/erv154 (PMC4449554; doi:10.1093/jxb/erv154)
Supplement: Supplementary Data [file supp_66_11_3417__index.html]

Differential effects of lesion mimic mutants in barley on disease development by facultative pathogens — Differential effects of lesion mimic mutants in barley on disease development by facultative pathogens — Supplementary Data 

# Differential effects of lesion mimic mutants in barley on disease development by facultative pathogens

## Supplementary Data

Data files

**Files in this Data Supplement:**

- Supplementary Data - Supplementary Data
