# Supplementary material for: Differential effects of lesion mimic mutants in barley on disease development by facultative pathogens
Source: J Exp Bot. 2015 Apr 8;66(11):3417–28. doi: 10.1093/jxb/erv154 (PMC4449554; doi:10.1093/jxb/erv154)
Supplement: Supplementary Data [file supp_erv154_jexbot145946_file001.pdf]

Differential effects of lesion mimic mutants in barley on disease development by facultative pathogens

Graham RD McGrann, Andrew Steed, Christopher Burt, Paul Nicholson, and James KM Brown

Supplemental Data

Fig. S1 Lesion mimic and Ramularia leaf spot infection phenotypes on leaves of Steptoe, nec1 (FN085), nec8 (FN303) and nec9 (FN227, FN364, FN450) mutant plants.

Fig. S2 Effects of independent mlo mutations in different barley genetic backgrounds on Fusarium culmorum lesion development.

Fig. S3 A proposed network of interaction between NEC1 and MLO and their effects on several traits. Summary of the effects of the (a) nec1 MLO, (b) NEC1 mlo-5¬, (c) nec1 mlo 5 and (d) NEC1 MLO ror genotypes on the traits reported in this paper. Thick diagonal cross: loss-of-function mutation. Arrow with solid edges: increase or decrease relative to wild-type MLO NEC1 plants. Solid arrow in (c): larger increase than in (b). Arrow with dashed edges: smaller change than in (a). Circled nc: no significant change from wild-type. No information is available for the difference in mlo-dependent cell death between NEC1 MLO and nec1 MLO plants (c). Note that in (d) the effect on F. culmorum lesion development was only tested in the NEC1 MLO ror1-2 mutant.

Table S1 qRT-PCR primers used in this study.

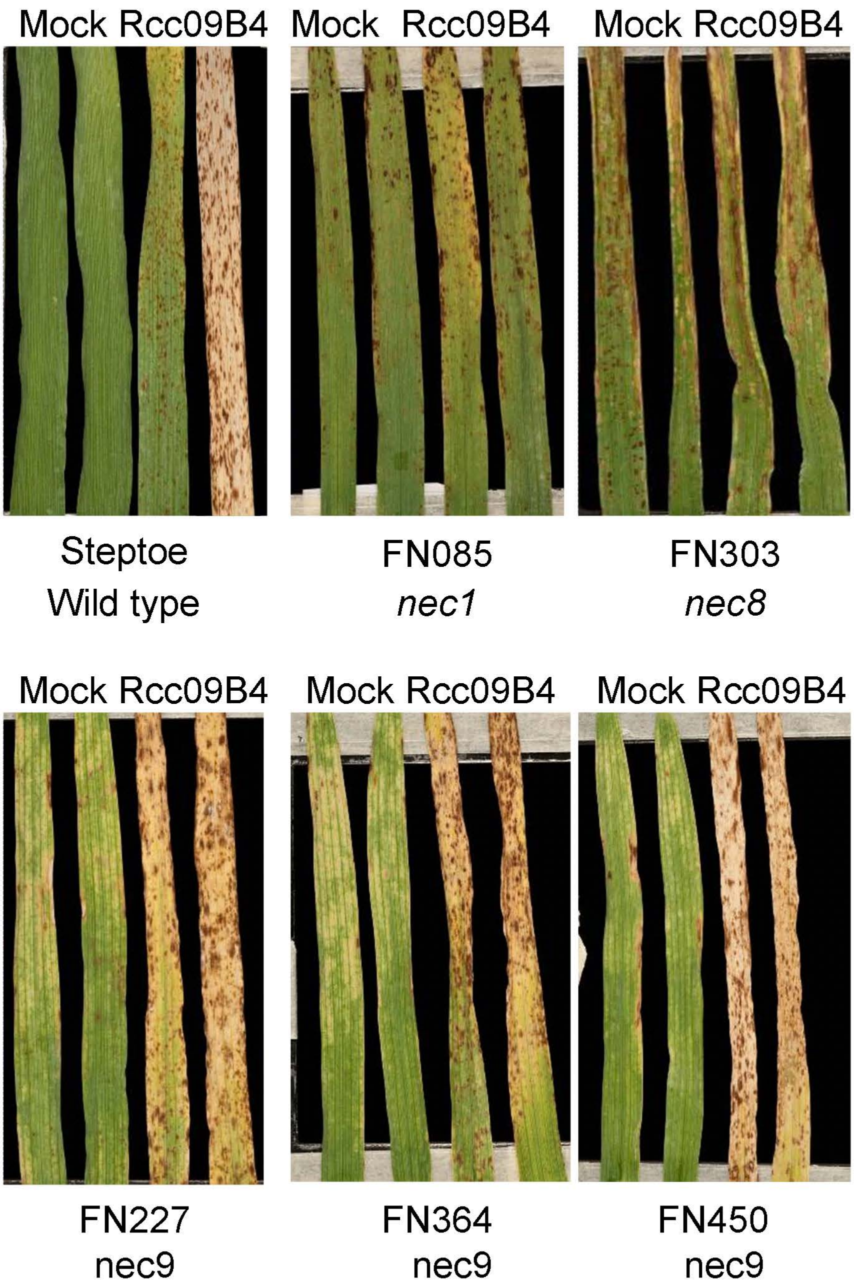
Mock Rcc09B4
Mock Rcc09B4
Mock Rcc09B4
Steptoe
Wild type
FN085
nec1
FN303
nec8
Mock Rcc09B4
Mock Rcc09B4
Mock Rcc09B4
FN227
nec9
FN364
nec9
FN450
nec9

Figure S1

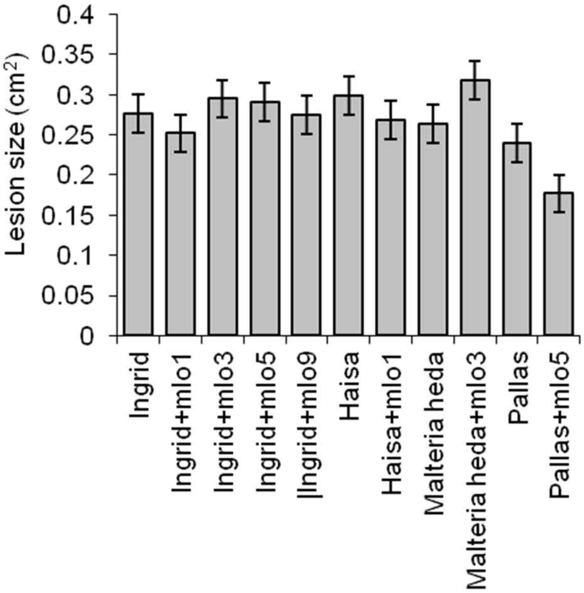
Lesion size (cm2)
0.4
0.35
0.3
0.25
0.2
0.15
0.1
0.05
0
Ingrid
Ingrid+mlo1
Ingrid+mlo3
Ingrid+mlo5
|Ingrid+mlo9
Haisa
Haisa+mlo1
Malteria heda
Malteria heda+mlo3
Pallas
Pallas+mlo5

Figure S2

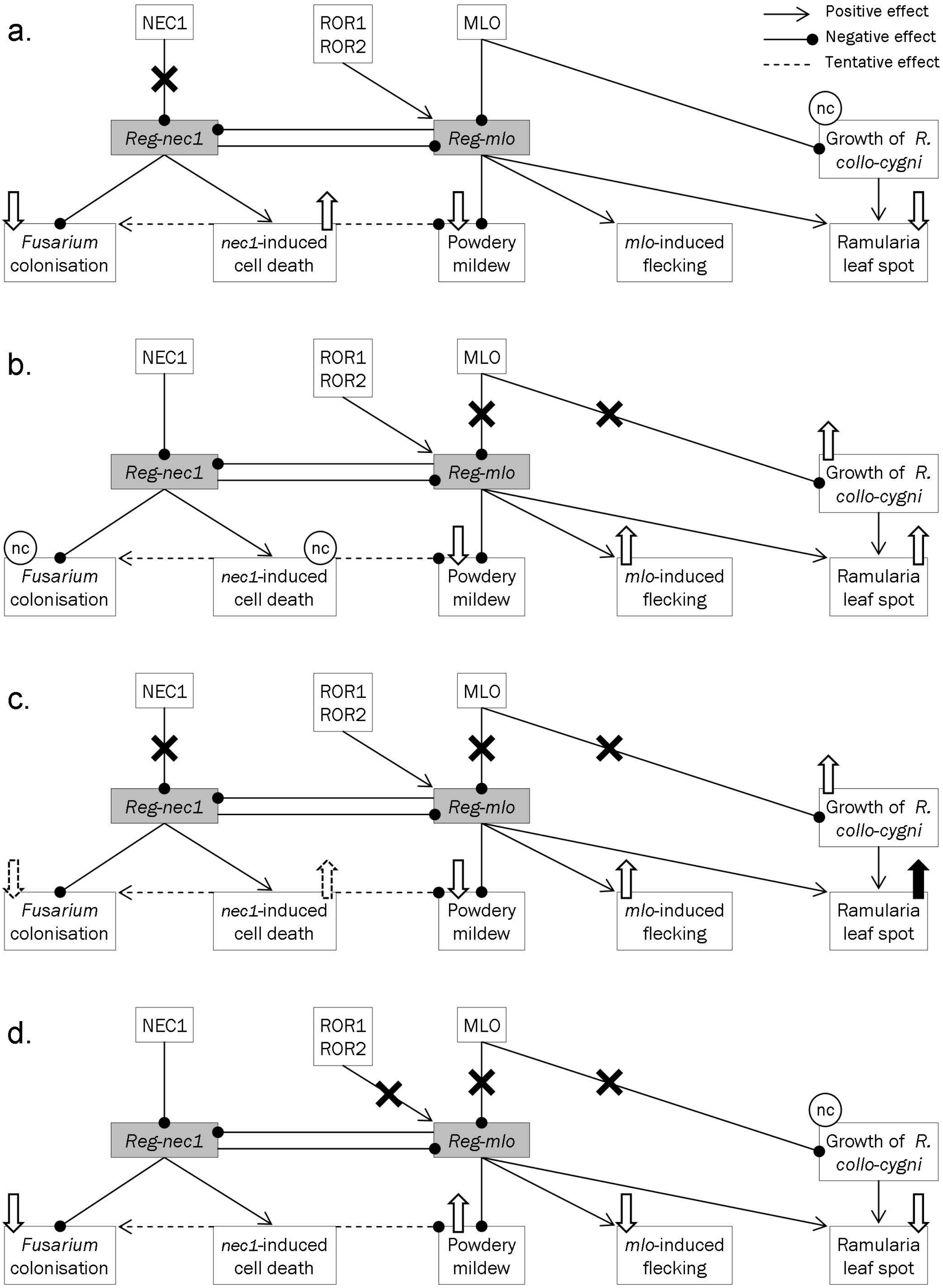
a.
b.
c.
d.
NEC1
ROR1
ROR2
MLO
Positive effect
Negative effect
Tentative effect
Reg-nec1
Reg-mlo
nc
Growth of R. collo-cygni
Fusarium colonisation
nec1-induced cell death
Powdery mildew
mlo-induced flecking
Ramularia leaf spot

Figure S3

Table S1 qRT-PCR primers used in this study

Target	Forward primer	Reverse primer	Amplicon size (bp)
HvPR1	AGCACGAAGCTGCAGGCGTA	TCTCGTCCACCCACAGCTTCAC	160 [b]
HvBI-1	GGCAGCTTCATGTTTGAGGT	AGGGCGTGCTTGATGTAGTC	122
HvCAT1	CCCGTCTGGAACAACAAC	CCCCGTGCATGAACAAC	134
HvCAT2	CGACGACAAGATGCTGCAGT	TGGTTGTTCTTGAAGCCGC	122 [a]
HvGPX1	AACGGCAACAATGTTTCTCC	ACAACGTGACCCTCCTTGTC	119
HvGPX2	ACGTGAATGGCAACAATGCT	ATGACATGCCCCTCTTTGTC	124
HvGR1	GGGGCTATAGTGGTCGATGA	AATGCTCCACCTTCCATCAG	116
HvAPX1	CGGAGCTTTTGAGTGGTGACA	CCGCAGCATATTTCTCCACAA	107 [a]
HvAPX2	CGCCGAGAAGAACTGC	GCCGGTCTTGGTGGC	82
HvCSD1	ACCTCGGAAATGTGACAGC	ACCCTTGCCAAGATCATCAG	140
Reference genes			
HvCyclophilin	TTGAGGACGAGATAAGGCCAG	GCGACTGACAAGGTGCAAGAG	120 [b]
HvEF1a	ATGATTCCCACCAAGCCCAT	ACACCAACAGCCACAGTTTGC	101 [b]
HvGAPDH	CCTTCCGTGTTCCCACTGTTG	ATGCCCTTGAGGTTTCCCTC	124 [b]
HvTUBA	AGTGTCCTGTCCACCCACTC	AGCATGAAGTGGATCCTTGG	248 [c]
HvUbiquitin	GCCGCACCCTCGCCGACTAC	CGGCGTTGGGGCACTCCTTC	219 [c]

[a] Shagimardova et al. (2010), [b] McGrann et al. (2009), [c] Colebrook et al. (2012)
